# Multidrug-resistant enterobacteria colonize commercial day-old broiler chicks in Nigeria

**DOI:** 10.14202/vetworld.2019.418-423

**Published:** 2019-03-16

**Authors:** Obianuju Nkiruka Okorafor, Madubuike Umunna Anyanwu, Emmanuel Onyeka Nwafor, George Nnamdi Anosa, Rita Ijeoma Udegbunam

**Affiliations:** 1 Department of Veterinary Medicine, University of Nigeria, Nsukka, Nigeria; 2 Department of Veterinary Pathology and Microbiology, University of Nigeria, Nsukka, Nigeria; 3 Department of Veterinary Surgery, University of Nigeria, Nsukka, Nigeria

**Keywords:** avian, coliforms, day-old chicks, *Enterobacteriaceae*, multidrug resistant

## Abstract

**Aim:**

This study was conducted to isolate generic enterobacteria from day-old broiler chicks in Nigeria, determine the antibacterial resistance profile, and assess multidrug resistance.

**Materials and Methods:**

The birds were sourced from five purposively-selected hatcheries (identified as A, B, C, D and E) in Southwest Nigeria. Non-duplicate cloacal swabs were collected from a total of 75 (15 birds per hatchery) randomly selected apparently healthy birds. Sampling was done in three batches of five chicks per batch at 2-week interval. Isolation of enterobacteria was done using MacConkey agar. The resistance of the isolates was determined using the disk diffusion method.

**Results:**

Of 15 processed samples of birds from each hatchery, all samples from hatcheries B, D, and E, 10 (66.7%) and 14 (93.3%) samples from hatcheries A and C, respectively, yielded pure cultures of *Escherichia* *coli*. *Klebsiella* was also isolated from 1 (7.1%) of the 14 and 2 (13.2%) of the 15 growth-positive samples from hatcheries C and D, respectively. The range of resistance among *E*. *coli* isolates was tetracycline (86.7-100%), ampicillin (80-100%), gentamicin (60-85.7%), sulfamethoxazole-trimethoprim (46.7-92.9%), enrofloxacin (40-100%), ciprofloxacin (26.7-86.7%), streptomycin (10-80%), cefotaxime (26.7-73.3%), amoxicillin-clavulanic acid (13.3-60%), and ceftazidime (6.7-40%). *Klebsiella* and all *E*. *coli* isolate from chicks of hatcheries B, C, and E, 80 and 93.3% of those from chicks of hatcheries A and D, respectively, exhibited resistance to three or more classes of antibacterial agents.

**Conclusion:**

Commercial day-old broiler chicks in Nigeria are colonized by multidrug-resistant coliforms (*E*. *coli* and *Klebsiella*) and are potential reservoirs and disseminators of these organisms.

## Introduction

The burgeoning Nigerian population (currently estimated to be 180 million with 3% annual growth rate) has resulted in unprecedented demand for animal food, of which the supply is far below the demand [1]. The poultry industry is the key livestock sector providing about 25% of animal protein and huge employment opportunities for Nigerians, with the sector increasing from 150,700 million chickens in 2005 to 192,313 million in 2010 [1-4]. In 2011, Nigeria ranked 19^th^ top hen egg producer in the world and top hen egg producer in Africa with total hen egg production of 636,000 metric tonnes valued at $527.49 million [1,5]. In Nigeria, day-old chicks (DOCs) are sourced mostly from hatcheries in the Southwestern region of the country [1]. Colonization of the gut by microbiota in young animals, including broiler chicks, occurs simultaneously with the development of the gut tissues [6,7]. Members of the family *Enterobacteriaceae* are among the microbiota colonizing the intestinal tract of chicks, protecting against pathogenic strains through competitive exclusion [7-11]. The hot humid tropical climate of Nigeria favors the growth of enterobacteria [1]. Apart from Newcastle disease and Avian influenza and perhaps global economic crises and inadequate credit, enterobacterial diseases such as colibacillosis and salmonellosis constitute the major constraint to poultry production in Nigeria [1,12,13]. These diseases are responsible for heavy morbidity and mortalities, especially at early stages of broiler chicks’ life [14]. Poultry farmers in Nigeria add massive amounts of different types/classes of antibacterials, including the critically important ones, in feed and/or drinking water to prevent these diseases [15,16]. Most times, this prevention strategy fails and the birds come down with the diseases [15]. Most antibacterials used in treating diseases in poultry are now ineffective; thus, cases of therapeutic failure and increased mortality are often reported in Nigeria [4,15]. This suggests that multidrug-resistant (MDR) bacteria might be offenders in most of these cases.

With the increasing reports of MDR enterobacteria in poultry and poultry meat worldwide, and the impact of these organisms on public health, there is increased interest in the origin of these organisms in the poultry production chain [9,11,17]. There is increasing number of reports on isolation of MDR enterobacteria from broilers in Nigeria [18,19]. Studies have shown that MDR enterobacteria could be pseudovertically transmitted through contaminated eggshell to DOCs and/or transovarianly from parent stock to hatchery [9,11,20,21]. Reports also showed that vertical transmission of MDR enterobacteria from parent stock to chicks from the top of production pyramid resulted in introduction and spread of resistance genes in poultry [22,23]. Parent stocks of commercial day-old broiler chicks in Nigeria are often sourced from overseas [1] and there are evidences that antibacterial agents, including critically important ones (such as extended-spectrum cephalosporins), are often injected *in ovo* in these countries [24,25]. In Nigeria, newly hatched chicks are vaccinated (usually with viral vaccines) in hatcheries and transported to production farms; they are considered “day old” until placed in production farms which are putative sources of resistant bacteria [26]. Assessing the potential of commercial day-old broiler chicks as reservoirs of resistant enterobacteria is crucial for devising strategies for improving biosecurity in hatcheries and parent stock farms, and for sourcing of parent stocks and DOCs. Determination of the antibiogram of enterobacteria colonizing healthy DOCs is important for adequate empiric treatment in these birds [14].

The spread of MDR enterobacteria in poultry industry is a matter of serious concern due to their zoonotic importance [11,14,27]. This is particularly important in Nigeria where poultry farming systems are not organized, and antibacterials are used to counterbalance poor hygiene and inadequate biosecurity [1]. The presence of MDR enterobacteria in day-old broiler chicks constitutes a threat to public health because these organisms could be acquired through direct and indirect contact with carrier birds, and/or consumption of undercooked poultry meat and associated products from the future broilers [11,14,21]. This is of great concern in Nigeria where there is no pre-slaughter assessment of antibacterial usage in broilers and some butchers consume raw chicken meat. Farmers and veterinarians are at greater risk of acquiring resistant organisms from DOCs due to frequent direct contact with these birds [14]. Passage of these organisms into the food chain/water bodies through poultry manure used in fertilizing farmlands is also of concern. This is because acquisition of these resistant organisms by humans and animals jeopardizes antimicrobial therapy in infected/carrier individuals [28]. Colonized individuals potentially serve as reservoirs and disseminators of these organisms to the environment and the public [11,14]. Thus, there is a need to assess antibacterial resistance profile of enterobacteria harbored by DOCs in Nigeria. Such information will be useful in devising strategy for controlling the development of antibiotic resistance in the poultry industry in Nigeria. Studies [9,10,14,29-34] in some countries showed that healthy and diseased DOCs are potential reservoirs of MDR enterobacteria. In Nigeria, a study [35] isolated antibacterial-resistant enterobacteria from dead-in-shell embryos while another [15] recovered resistant enterobacteria in clinical samples of DOCs. No study has assessed the potential of apparently healthy commercial day-old broiler chicks in Nigeria as reservoirs of MDR enterobacteria, whereas these birds are important source of the introduction of MDR organisms in broiler production chain. Surveillance of resistance in commensal enterobacteria is important because they are potential reservoirs of resistance genes and more ubiquitous than pathogens [14].

The objective of this study was to isolate enterobacteria colonizing commercial day-old broiler chicks and to determine the antibacterial and multidrug resistance profiles of the isolates.

## Materials and Methods

### Ethical approval

Permission to conduct the study and ethical clearance was obtained from the Medical Research Ethics Committee of the University of Nigeria, Nsukka. All the birds in this study were handled humanely in accordance with the Helsinki Declaration [36].

### Sampling

This cross-sectional study was conducted between January and August 2016, in Nsukka, Southeast Nigeria. Five hatcheries (identified as A, B, C, D and E) in Southwest Nigeria, mostly patronized by DOCs distributors and farmers in the Southeastern region of the country were purposively selected for this study. Immediately on arrival, after overnight transport, five birds were randomly selected from every batch of 100 DOCs sourced from each of the hatcheries. The sampling was done 3 times at 2-week interval to increase the chances of recovering resistant organisms if there is contamination over time. A total of 15 birds were sampled per hatchery. Selected chicks were placed in a new carton obtained from the respective hatchery and transported to the Veterinary Microbiology Laboratory, University of Nigeria, Nsukka, within 5 min. Non-duplicate cloacal swabs were collected from each of the bird using a sterile swab stick.

### Isolation and generic identification of enterobacteria from day-old broiler chicks

The swabs were cultured directly on MacConkey agar and incubated at 37°C for 24 h in ambient air. Morphotypes were described and recorded appropriately. For mixed cultures, isolates were purified by subculturing on MacConkey agar and incubated at 37°C for 24 h. Phenotypic characterization of the isolates to generic level was done by subjecting them to various tests such as Gram-staining, citrate, indole, methyl red, ornithine decarboxylase, triple sugar iron agar test, and subculturing on eosin methylene blue agar following standard procedures [37].

### Determination of antibiogram of generic enterobacterial isolates from day-old broiler chicks

Antibacterial resistance/susceptibility profiles of the enterobacterial isolates were determined by the disk diffusion method using 10 antibacterial agents belonging to six classes: Fluoroquinolones – ciprofloxacin (CIP; 5 µg) and enrofloxacin (ENR; 5 µg), folate pathway antagonists – sulfamethoxazole-trimethoprim (SXT; 25 µg), β-lactams/cephalosporins – ampicillin (AMP; 10 µg), ceftazidime (CTZ; 30 µg), and cefotaxime (CTX; 30 µg), β-lactam combination agents – amoxicillin-clavulanic acid (AMC; 20/10 µg), tetracycline (TET; 30 µg), aminoglycosides – gentamicin (GEN; 10 µg) and streptomycin (STR; 10 µg). *Escherichia* *coli* American Type Culture Collection^®^ (ATCC) 25922 and *Klebsiella pneumoniae* ATCC 700603 were used as reference strains. Results of the antibacterial resistance/susceptibility testing were interpreted according to the Clinical and Laboratory Standards Institute [38] guidelines for *Enterobacteriaceae*. Intermediately susceptible isolates were classified as resistant in this study. An isolate resistant to agents in three or more classes/categories was considered MDR [39].

### Statistical analysis

The frequencies of the occurrence of enterobacterial genera and resistance of the isolates from pooled samples of birds from each hatchery were entered into Microsoft Excel version 2010 (Microsoft Corporation, Redmond, USA) and their percentages calculated. The median value of resistance to the antibacterial agents was determined using Microsoft Excel.

## Results

### Isolation rate of generic enterobacteria from day-old broiler chicks

Of 15 processed samples from each hatchery, all from hatcheries B, D, and E, 10 (66.7%) and 14 (93.3%) from hatcheries A and C, yielded only pure cultures of *E. coli*. Of the 14 and 15 positive samples from hatcheries C and D, 1 (7.1%) and 2 (13.3%) also yielded *Klebsiella*, respectively.

### Antibiogram of generic enterobacterial isolates from day-old broiler chicks

Antibacterial resistance profiles of *E*. *coli* isolates from various hatcheries are presented in Table-1. Of the 10 *E*. *coli* isolates from chicks of hatchery A, 90% were resistant to TET and AMP, 10% to STR, 60% to GEN, AMC, CTX, and ENR, and 30% to CTZ and 40% to CIP. Of the 15 *E*. *coli* isolates from chicks of hatchery B, all were resistant to TET and AMP, 46.7% to SXT, 40% to STR, 60% to GEN, 40% to AMC, 6.7% to CTZ, 26.7% to CTX and CIP, and 66.7% to ENR. Among the 14 *E*. *coli* isolates from chicks of hatchery C, all were resistant to AMP and ENR, 92.9% to SXT and TET, 28.6% to STR and CTZ, 85.7% to GEN and CIP, 21.4% to AMC, and 57.1% to CTX. Among the 15 *E*. *coli* isolates from chicks of hatchery D, 60% were resistant to SXT and GEN, 86.7% to TET, 26.7% to STR, 80% to AMP, 20% to AMC, 6.7% to CTZ, and 40% to CTX, ENR, and CIP. Of the 15 *E*. *coli* isolates from chicks of hatchery E, all were resistant to TET and AMP, 86.7% to SXT and CIP, 80% to STR and GEN, 13.3% to AMC, 40% to CTZ, 73.3% to CTX, and 93.3% to ENR. The range of resistance among *E*. *coli* isolates from the hatcheries was TET (86.7-100%, median = 92.9%), AMP (80-100%, median = 100%), GEN (60-85.7%, median = 85.7%), SXT (46.7-92.9%, median = 92.9%), ENR (40-100%, median = 100%), CIP (26.7-86.7%, median = 85.7%), STR (10-80%, median = 28.6%), CTX (26.7-73.3%, median = 57.1%), AMC (13.3-60%, median = 21.4%), and CTZ (6.7-40%, median = 28.6%).

**Table-1:** Antibiogram of *Escherichia coli* isolates from day-old broiler chicks from different hatcheries.

Antibacterial agent (concentration)	Percentage of resistant isolates per hatchery				

	A (n=10)	B (n=15)	C (n=14)	D (n=15)	E (n=15)
Sulfamethoxazole-trimethoprim (25 μg)	0	46.7	92.9	60	86.7
Tetracycline (30 μg)	90	100	92.9	86.7	100
Ampicillin (10 μg)	90	100	100	80	100
Ceftazidime (30 μg)	30	60.7	28.6	6.7	40
Cefotaxime (30 μg)	60	26.7	57.1	40	73.3
Amoxicillin-clavulanic acid (20/10 μg)	60	40	21.4	20	13.3
Streptomycin (10 μg)	10	40	28.6	26.7	80
Gentamicin (10 μg)	60	60	85.7	60	80
Enrofloxacin (5 μg)	60	66.7	100	40	93.3
Ciprofloxacin (5 μg)	40	26.7	85.7	40	86.7

All *E*. *coli* isolates from chicks of hatcheries B, C, and E, 80% and 93.3% of those from hatcheries A and D, respectively, exhibited resistance to at least a drug in three or more classes of antibacterial agents and thus MDR (Table-2). Both *Klebsiella* isolates from chicks of hatchery D were resistant to CTX, TET, and SXT (that is, three classes of antibacterial agents) while 1 (50%) was resistant to AMC, GEN, AMP, CIP, ENR, and CTZ (that is, four classes of antibacterial agents). *Klebsiella* isolate from a chick of hatchery C was susceptible to only STR but exhibited resistance to all the six classes of antibacterial agents tested.

**Table-2:** Resistance of *Escherichia coli* isolates from day-old broiler chicks from different hatcheries to antibacterial classes.

Number of antibacterial class	Percentage of resistant isolates per hatchery				

	A (n=10)	B (n=15)	C (n=14)	D (n=15)	E (n=15)
1	0	0	0	6.7	0
2	20	0	0	0	0
≥ 3	80	100	100	93.3	100

## Discussion

The fact that only *E*. *coli* was isolated from all the samples from three hatcheries (B, D, and E) and most of those from the remaining two hatcheries (A and C) in this study suggested that *E*. *coli* may be the dominant enterobacteria colonizing commercial day-old broiler chicks in Nigeria. Less frequent isolation of *Klebsiella* in this study suggested that it may not be a common enterobacteria genus colonizing commercial day-old broiler chicks in Nigeria. Members of the family *Enterobacteriaceae* are known to colonize DOCs even before they have contact with the environment [7,11]. Elsewhere, investigators isolated *E*. *coli* [29,30-32,40] and *Klebsiella* [29,34] from samples of healthy DOCs. In the presented experiment, direct plating was employed in culturing the samples; this may account for why other enterobacteria genera such as *Salmonella* which requires pre-enrichment/enrichment could not be isolated. The previous studies [31,41] elsewhere reported isolation of *Salmonella* from samples of DOCs, but Nazer *et al*. [29] did not isolate *Salmonella* from 120 healthy day-old broiler chicks despite pre-enrichment and enrichment processes. Therefore, it is also possible that the absence of *Salmonella* in this present study may be because there was no contamination of the eggs during formation in the oviduct, during or after oviposition [42]. Chicks are hatched with sterile intestinal tract, but the microbial populations of DOCs (chicks that have neither eaten nor placed in production farm) vary due to differences in bacteria ingestion from hatching debris, environment, and production facility [26].

The moderate to high rate of resistance exhibited by isolates in this study suggested selection probably due to acquisition of genes encoding resistance to the drugs. The AMP resistance (80-100%) among *E*. *coli* isolates in this study is higher than 70% AMP resistance among *E*. *coli* isolates from DOCs in Spain [14]. Das *et al*. [32] reported 82.98% AMP resistance among *E*. *coli* isolates from DOCs in Bangladesh, this result is within the AMP resistance range (80-100%) but lower than the median value (100%) in this study. Jiménez-Belenguer *et al*. [14] reported 30% TET resistance, a finding that is lesser than that (86.7-100%) of this study while Das *et al*. [32] recorded 100% TET resistance which is comparable to the result (80-100%, median = 100%) of this study. Zibandeh *et al*. [43] recorded 67% TET resistance among *E. coli* isolates from 1-day-old chicks in Iran; this finding is lower than the result (86.7-100%) of this study. Jiménez-Belenguer *et al*. [14] reported <10% STR and GEN resistances, whereas Das *et al*. [32] did not observe GEN resistance; these findings contrasted the results of this study. Das *et al*. [32] reported 14.89% ENR resistance which is lower than the finding (40-100%) of this study. Nazer *et al*. [29] and Abdi-Hachesoo *et al*. [30] observed 48.97 and 41.9% ENR resistance among *E*. *coli* isolates from day-old chicks in Iran, respectively; these results are in the range (40-100%) of ENR resistance but lower than the median value (100%) in this study. Abdi-Hachesoo *et al*. [30] also reported 36.2% CIP resistance which is in the range (26.7-86.7%) of CIP resistance but lower than the median value (85.7%) in this study, whereas Jiménez-Belenguer *et al*. [14] and Das *et al*. [32] reported 16 and 25.53% CIP resistance, respectively, which are lower than the result of this study. Biswas *et al*. [31] reported that *E*. *coli* isolates from day-old broiler chicks in Bangladesh exhibited high susceptibility to GEN, moderate susceptibility to CIP, and less susceptibility to TET. Variation in resistance among isolates in these studies could be related to the differences in susceptibility testing method(s) employed, poultry management, level of contamination of hatcheries from where the birds were sourced, and usage of antibacterials in the study areas. In this study, the medical history of the parent stock of the sampled chicks and antibacterial usage in the various hatcheries were not traced.

The fact that 80-100% of *E*. *coli* and *Klebsiella* isolates in this study were MDR (exhibited resistance to agents in three or more classes [39]) indicated that the birds are potential reservoirs of these organisms [14,44]. This finding suggested that the isolates could have acquired genes encoding resistance to the various antibacterials probably due to selection pressure [14,19]. This finding of resistance to many classes of antibacterial suggested that these agents have been tremendously abused in Nigerian poultry industry due to uncontrolled use of antimicrobials in the country [19].

The finding of multidrug resistance in this study calls for serious concern due to limited option for empiric therapy if the birds become infected with these organisms [12,14,30]. This could result in large-scale mortality and resorting to the use of last-line antibacterials such as colistin for the prevention and treatment of poultry diseases in Nigeria. Unfortunately, there is now a described plasmid-mediated colistin resistance (with different genes that have been described to date) [45]. Moreover, these organisms pose a serious threat to the health of individuals that acquire them on making direct contact (such as poultry farmers, veterinarians, and consumers of meat and associated products derived from these future broilers) with the birds. These organisms could be transmitted to the public through the primary contacts and/or poultry manure used in fertilizing farmlands and subsequently contaminating the food chain [11,14,30]. In this study, usage of antibacterials in the parent stock and hatcheries were not traced. However, possible sources of MDR organisms in the sampled birds include: Contaminated parent stock, egg surfaces, hatcheries/hatchery workers, and/or during transportation [14,20,25,34].

## Conclusion

*E*. *coli* is the dominant genus of enterobacteria colonizing commercial day-old broiler chicks in Nigeria. High percentages (80-100%) of commercial day-old broiler chicks in Nigeria are colonized by MDR enterobacteria. Thus, these birds are potential reservoirs and disseminators of these organisms and genes encoding resistance to different classes of antibacterial agents. This has a tremendous impact on the food chain and epidemiology of antibacterial resistance. Therefore, in Nigeria, the acquisition of broiler parent stock should be done with caution and attention should be paid on the use of antibacterials in the poultry industry. However, further studies to determine the resistance genes harbored by the isolates are recommended.

## Authors’ Contributions

ONO, GNA, and RIU conceptualized the study. ONO, MUA, and EON collected and processed the samples. All authors participated in draft and revision of the manuscript. All authors read and approved the final manuscript.
